# Tamoxifen is a candidate first‐in‐class inhibitor of acid ceramidase that reduces amitotic division in polyploid giant cancer cells—Unrecognized players in tumorigenesis

**DOI:** 10.1002/cam4.2960

**Published:** 2020-03-05

**Authors:** Shai White‐Gilbertson, Ping Lu, Christian M. Jones, Stephanie Chiodini, Deborah Hurley, Arabinda Das, Joe R. Delaney, James S. Norris, Christina Voelkel‐Johnson

**Affiliations:** ^1^ Department of Microbiology & Immunology Medical University of South Carolina Charleston SC USA; ^2^ Department of Biochemistry and Molecular Biology Medical University of South Carolina Charleston SC USA; ^3^ South Carolina Central Cancer Registry SCDHEC Columbia SC USA; ^4^ Department of Neuroscience Medical University of South Carolina Charleston SC USA

**Keywords:** acid ceramidase, cancer, PGCC offspring, polyploidy, radiation therapy, recurrence, sphingolipid, tamoxifen

## Abstract

Polyploid giant cancer cells (PGCC) represent a poorly understood, small subpopulation of tumor cells that are increasingly being recognized for their critical role in therapy resistance, metastasis, and cancer recurrence. PGCC have the potential to generate progeny through primitive or cleavage‐like division, which allows them to evade antimitotic insults. We recently demonstrated that the sphingolipid enzyme acid ceramidase (ASAH1) is required for this process. Since specific ASAH1 inhibitors are not clinically available, we investigated whether tamoxifen, which interferes with ASAH1 function via off‐target effects, has a potential clinical benefit independent of estrogen signaling. Our results show that tamoxifen inhibits generation of PGCC offspring in prostate cancer, glioblastoma, and melanoma cells. Analysis of two state‐level cancer registries revealed that tamoxifen improves survival outcomes for second, nonbreast cancers that develop in women with early stage breast cancer. Our results suggest that tamoxifen may have a clinical benefit in a variety of cancers that is independent of estrogen signaling and could be due to its inhibition of acid ceramidase. Thus the distinct application of tamoxifen as potentially a first‐in‐class therapeutic that inhibits the generation of PGCC offspring should be considered in future clinical trials.

## Graphical abstract

This study suggests that tamoxifen has an estrogen‐signaling independent clinical benefit in a variety of cancers that could be due to acid ceramidase inhibition. This would make tamoxifen a first‐in‐class therapeutic against formation of PGCC progeny.

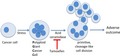

**Abbreviations**
ASAH1: acid ceramidase
PGCC: polyploid giant cancer cells

## 1 INTRODUCTION

Acid ceramidase (ASAH1) hydrolyzes the pro‐death sphingolipid ceramide and plays a critical role in driving recurrence in prostate cancer and in supporting tumor‐initiating cells in glioblastoma and melanoma.^1^, ^2^, ^3^ While ASAH1 appears to be crucial in subpopulations of cells that seed tumors, the underlying reason is not fully elucidated. ASAH1 is required during the 2‐4 cell blastomere stage during embryonic development but is largely dispensable in somatic cells as demonstrated by nearly phenotype‐free conditional knockout mice.^4^, ^5^ The requirement for ASAH1 during early embryonic development is intriguing as the blastomere stage is hypothesized to hold a key in the process of cancer resistance and recurrence.^6^, ^7^

Exposure of cancer cells to stress in the tumor microenvironment or in response to therapy results in reversion to a blastomere‐like stage characterized by the generation of polyploid giant cancer cells (PGCC) which store multiple nuclei that are subsequently utilized to generate progeny via budding instead of mitosis.^6^, ^8^ This asymmetric form of cell division produces a new generation of cancer cells that resume mitosis.^9^, ^10^, ^11^, ^12^ A recent study shows that generation of PGCC offspring is dependent on the expression of ASAH1, making this enzyme the first rational target to prevent the formation of dangerous PGCC progeny.^13^ Unfortunately, specific inhibitors of ASAH1 are not clinically available, which prompted the question of whether drugs with a known off‐target effect on ASAH1 might be repurposed.^14^, ^15^

Tamoxifen is commonly used for the treatment of estrogen receptor‐positive breast cancer in premenopausal women but is also approved or under evaluation for other conditions. This drug is included on the WHO List of Essential Medicines as one of the most effective and safe medicines needed in a health‐care system. We were interested in the reported off‐target effect by which tamoxifen inhibits ASAH1, which occurs as a consequence of increased lysosomal permeability followed by a time‐ and dose‐dependent downregulation of ASAH1 protein expression.^15^ In this study, we tested the hypothesis that tamoxifen exerts an inhibitory effect on generation of PGCC offspring through inhibition of ASAH1. Using prostate cancer, glioblastoma, and melanoma cells, which by definition are not derived from estrogen‐dependent cancers, we demonstrate that tamoxifen is highly effective in reducing PGCC progeny. We then evaluated cancer registry databases to identify a subset of younger breast cancer patients that developed a second cancer to ask whether tamoxifen had a positive impact on the nonbreast cancer. We found that tamoxifen provided a significant increase in survival for the second (nonbreast) cancer, which suggests the possibility that tamoxifen contributed to inhibition of ASAH1.

## 2 RESULTS

### 2.1 Treatment stress increases the formation of PGCC that generate offspring in a nonmitotic, ASAH1‐dependent manner

**FIGURE 1:**
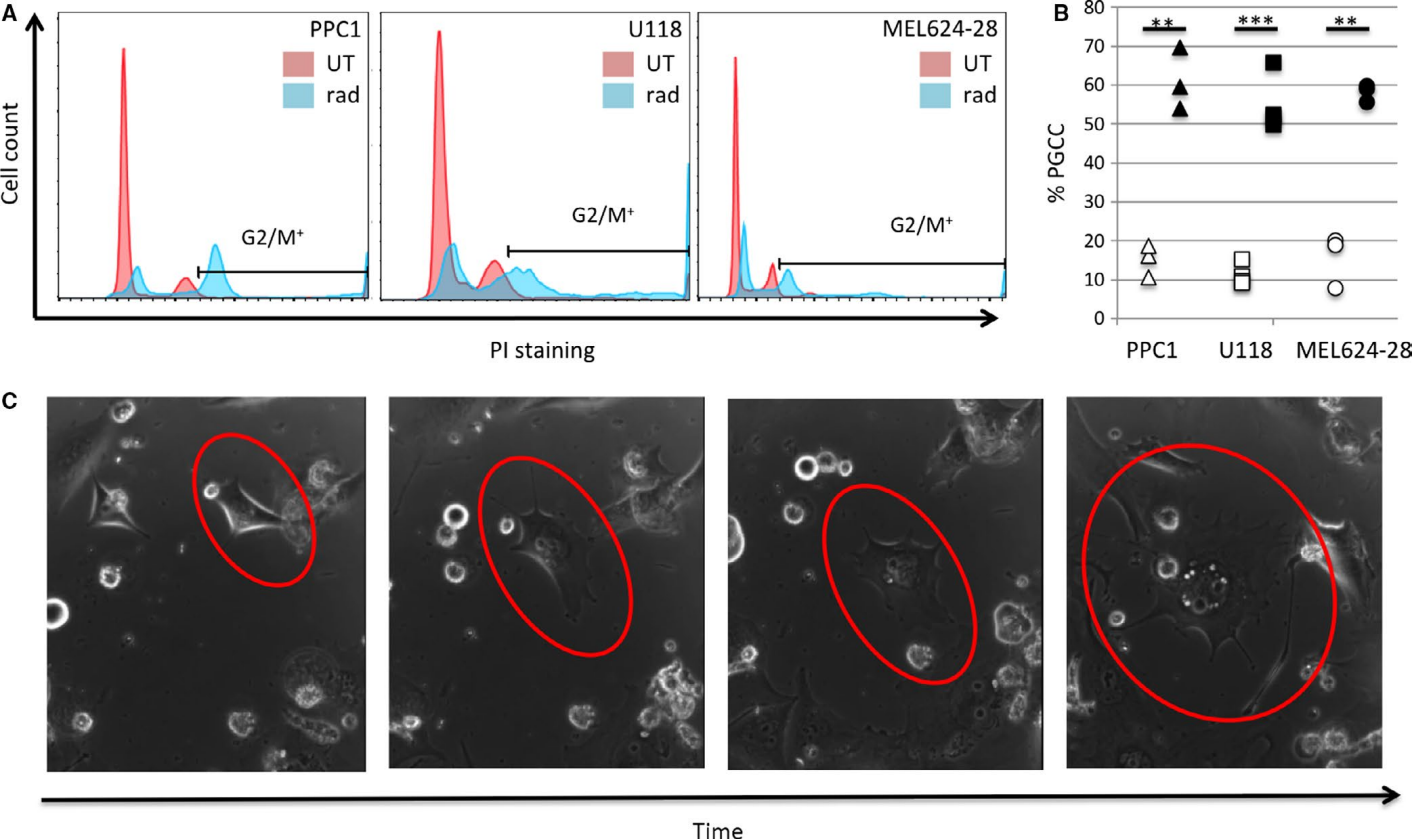
Radiation induces PGCC in prostate cancer, melanoma, and glioblastoma cells. (A) Using cell cycle analysis by flow cytometry, cells with propidium iodide staining greater than at G2 in baseline conditions were gated as “G2/M+.”. The percent of each analyzed population composed of polyploid cells, before and after irradiation, is quantified in (B), ^**^ *P* < .001, ^***^ *P* < .0001. (C) Time‐lapse images of a PPC1 cell undergoing enlargement through endoreplication. Full movie with scale bar provided in SM1

**FIGURE 2:**
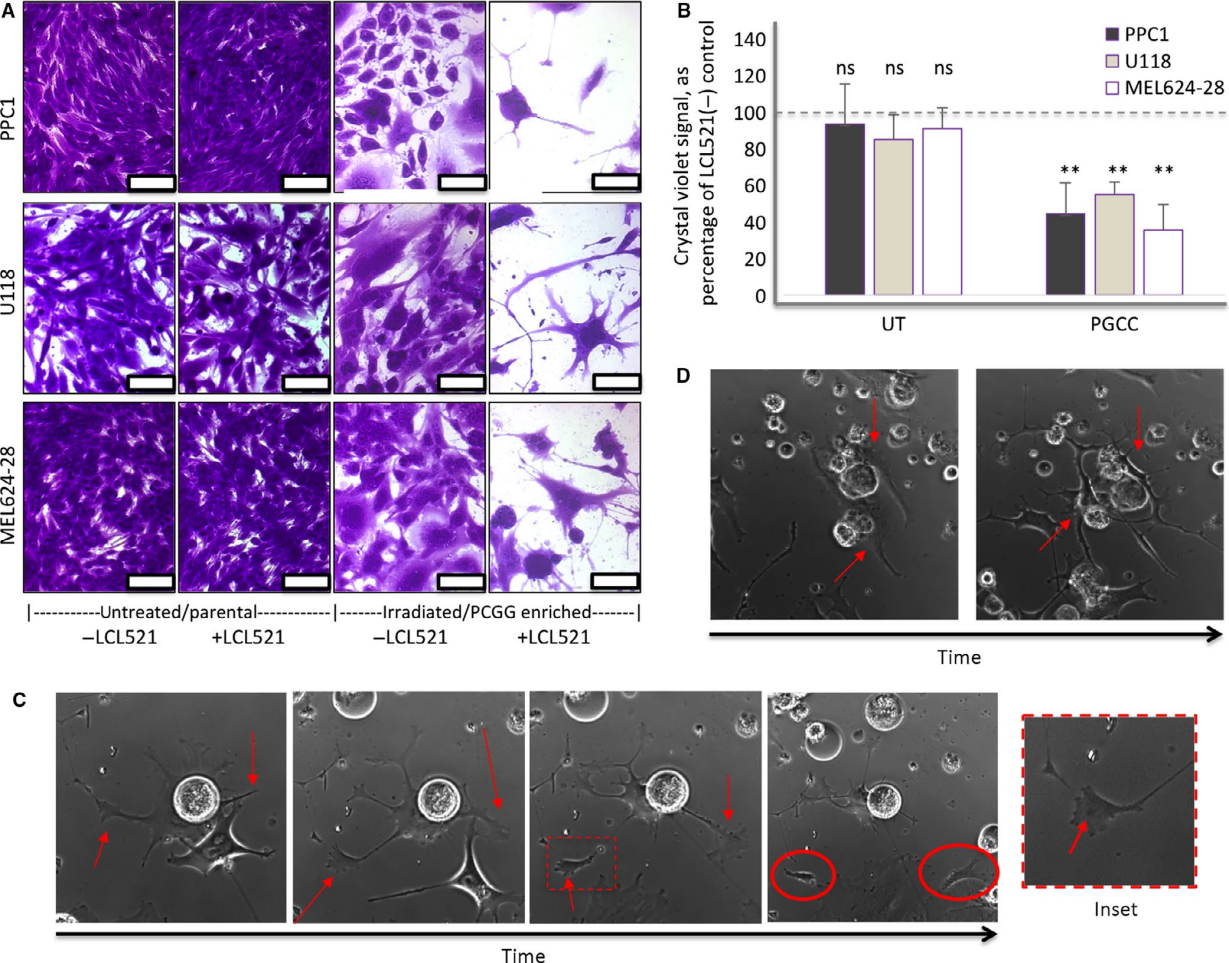
LCL521 inhibits formation of PGCC progeny in prostate cancer, melanoma, and glioblastoma cells. (A) Representative images of crystal violet‐stained untreated or irradiated cells expanded in the absence or presence of 5 µM LCL521. Scale bar = 100 µM. (B) Quantification of crystal violet staining from three independently performed recovery assays. Percentage staining in the presence of LCL521 was expressed relative to the untreated control (matching conditions without LCL521). LCL521 significantly inhibited recovery in radiation treated cells (PGCC) relative to untreated cells **P < .01. (C) Time‐lapse images of a PPC1 PGCC extruding two daughter cells over the course of 10 hours. Red arrows in the first three panels indicate budding sites. Red circles indicate daughter cells. Red arrow in the inset shows a single nucleus in a daughter cell. Full movie with scale bar in SM2. (D) Representative images from movie SM3, showing the “barren branchin” phenotype assumed by PGCC treated with LCL521. Red arrows show change from typical morphology to branched

Analysis of DNA content shows that cancer cells from various origins contain PGCC at baseline and that the percentage increases significantly following radiation stress (Figure 1A,B). The increase in PGCC following treatment is not solely due to a residual, therapy resistant population as absolute numbers of PGCC are also significantly elevated (Figure S1A). Furthermore, PGCC appear to develop due to endoreplication rather than fusion (Figure 1B; Movie S1). Treatment with the ASAH1 inhibitor LCL521 had no significant impact on parental (mitotic) populations (Figure 2A,B). PGCC generated by radiation produce offspring in a nonmitotic primitive cleavage‐like fashion as they recover from treatment stress, which is visualized through the release of small daughter cells (Figure 2C; Movie S2). Treatment with LCL521 to inhibit ASAH1 activity significantly reduced the generation of PGCC offspring following cessation of treatment stress (Figure 2A,B), which morphologically appeared as stunted or “barren branching” and is suggestive of a failure to extrude progeny (Figure 2A,D; Movie S3).

### 2.2 Tamoxifen inhibits ASAH1 activity and colony formation in PGCC‐derived from various cancer origins

**FIGURE 3:**
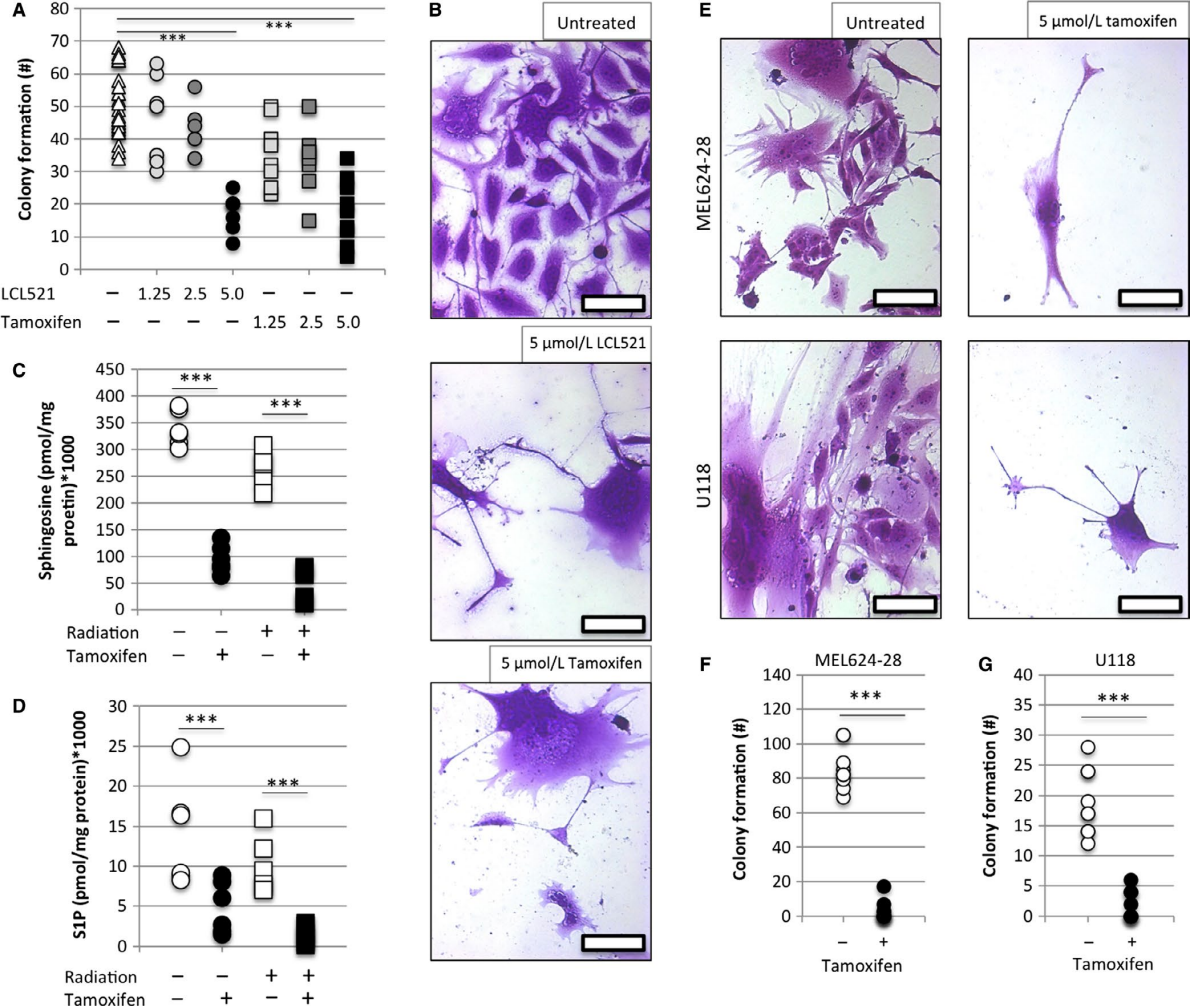
Tamoxifen inhibits ASAH1 activity and reduces formation of PGCC progeny in prostate cancer, melanoma, and glioblastoma cells. (A) Evaluation of LCL521 and tamoxifen dosages that inhibit colony formation in PPC1‐derived PGCC. ^***^ *P* < .0001. (B) Representative images of A, scale bar = 100 µM. (C, D) Lipidomics analysis of PPC1 cells treated with 5 µM tamoxifen for 5 hours. Data shown are triplicates from two independently performed experiments. (E) Representative images of F and G. (F, G) Colony formation assays in the absence and presence of 5 µM tamoxifen using MEL624‐28‐ or U118‐derived PGCC. ^***^ *P* < .0001

Tamoxifen is an established drug with decades of dosing and safety data. The typical dose of 20‐40 mg/day results in serum concentrations of 96‐200 ng/mL (0.25‐0.5 µM).^16^ Tissue accumulation, both for normal tissue and cancer tissue, is approximately 10‐fold higher than serum levels, suggesting that clinically relevant concentrations are between 2.5 and 5 µM.^17^, ^18^ Because tamoxifen was reported to exert an off‐target effect on ASAH1, we evaluated its activity on PGCC progeny and found that colony formation in PGCC derived from PPC1 was strongly inhibited at 5 µM tamoxifen with an efficacy similar to LCL521 (Figure 3A,B). Lipidomic analysis confirmed that tamoxifen significantly reduced intracellular levels of the ASAH1 product sphingosine as well as the downstream metabolite sphingosine‐1‐phosphate (Figure 3C,D). Tamoxifen also effectively inhibited colony formation in melanoma or glioblastoma‐derived PGCC (Figure 3E‐G).

### 2.3 Tamoxifen prescribed for breast cancer improves outcomes for nonbreast cancers

To determine if tamoxifen has a potential clinical benefit beyond its intended use as an inhibitor of estrogen signaling, data for a cohort of breast cancer survivors were extracted from the state central cancer registries of South Carolina and North Carolina. Our goal was to identify patients 30‐50 years in age at diagnosis of early stage breast cancer (SEER stage 0/1), since this population is unlikely to experience significant mortality from their cancer, and is generally prescribed tamoxifen if hormone therapy is considered appropriate (older, postmenopausal patients are prescribed aromatase inhibitors). Tamoxifen is typically taken for 5 years and since our goal was to examine the possible off‐target effects of the drug on the outcomes for nonbreast cancers, we selected the subset of women who developed a second cancer within four years of the original breast malignancy for further analysis. Patient characteristics in Table 1 demonstrate that the use of tamoxifen was not significantly different across race, age, stage, grade, and histology. In South Carolina, we were additionally able to calculate incidence rates of second malignancies in our study populations, which also did not differ significantly (*P* = .38). The incidence of developing a second (nonbreast) tumor in women not taking tamoxifen was 1.51% (102 of 6751 patients) and the rate in the tamoxifen group was 1.30% (46 of 3555 patients).

**TABLE 1:** Patient and disease characteristics for study populations (North and South Carolina)

	North Carolina			South Carolina		
	No tamox n = 124 (%)	Tamox n = 71 (%)	*P* value	No tamox n = 102 (%)	Tamox n = 46 (%)	*P* value
Race						
White	101 (81.4)	58 (81.7)		65 (63.7)	33 (71.7)	
All other	23 (18.6)	13 (18.3)	.967	37 (36.6)	13 (28.3)	.340
Breast cancer						
Diagnosis age						
30‐40	28 (22.6)	8 (11.3)		11 (10.8)	9 (19.6)	
41‐51	96 (77.4)	63 (88.7)	.050	91 (89.2)	37 (80.4)	.148
Grade						
1	13 (10.5)	12 (16.9)		19 (18.6)	15 (32.6)	
2	36 (29.0)	29 (40.8)		23 (22.6)	11 (23.9)	
3	46 (37.1)	18 (25.4)		45 (44.1)	11 (23.9)	
Other/unk	29 (23.4)	12 (16.9)	.105	15 (14.7)	9 (19.6)	.086
SEER stage						
In situ 0	36 (29.0)	19 (26.8)		30 (29.4)	11 (23.9)	
Localized 1	88 (71.0)	52 (73.2)	.734	72 (70.6)	35 (76.1)	.479
Histology						
Ductal carcinoma	94 (75.8)	49 (69.0)		74 (72.6)	31 (67.4)	
Other	30 (24.2)	22 (31.0)	.302	28 (27.4)	15 (32.6)	.522
Nonbreast cancer						
Diagnosis age						
30‐50	101 (81.4)	56 (78.9)		77 (75.5)	33 (71.7)	
51+	23 (18.6)	15 (21.1)	.662	25 (24.5)	13 (28.3)	.629
SEER stage						
In situ‐‐region 0‐2	77 (62.1)	47 (66.2)		63 (61.80029	33 (71.7)	
Distant/unk 3+	47 (37.9)	24 (33.8)	.567	39 (38.2)	13 (28.3)	.239
Histology						
Adenocarcinoma	37 (29.8)	30 (42.2)		25 (24.5)	8 (17.4)	
Melanoma	18 (14.5)	13 (18.3)		11 (10.8)	8 (17.4)	
Other	69 (55.6)	28 (39.4)	.090	66 (64.7)	30 (65.2)	.407
Surgery						
Yes	92 (74.2)	58 (81.7)		82 (80.4)	38 (82.6)	
No	32 (25.8)	13 (18.3)	.232	20 (19.6)	8 (17.4)	.750
Radiation						
Yes	18 (14.5)	11 (15.5)		17 (16.7)	7 (15.2)	
No	106 (85.5)	60 (84.5)	.854	85 (83.3)	39 (84.8)	.825
Chemotherapy						
Yes	32 (25.8)	15 (21.1)		30 (29.4)	14 (30.4)	
No	92 (74.2)	56 (78.9)	.462	72 (70.6)	32 (69.6)	.900

Note

Breast cancer survivors with tamoxifen exposure during a nonbreast malignancy were compared with those without tamoxifen. No significant differences were found between the patient groups (all *P* > .05).

**FIGURE 4:**
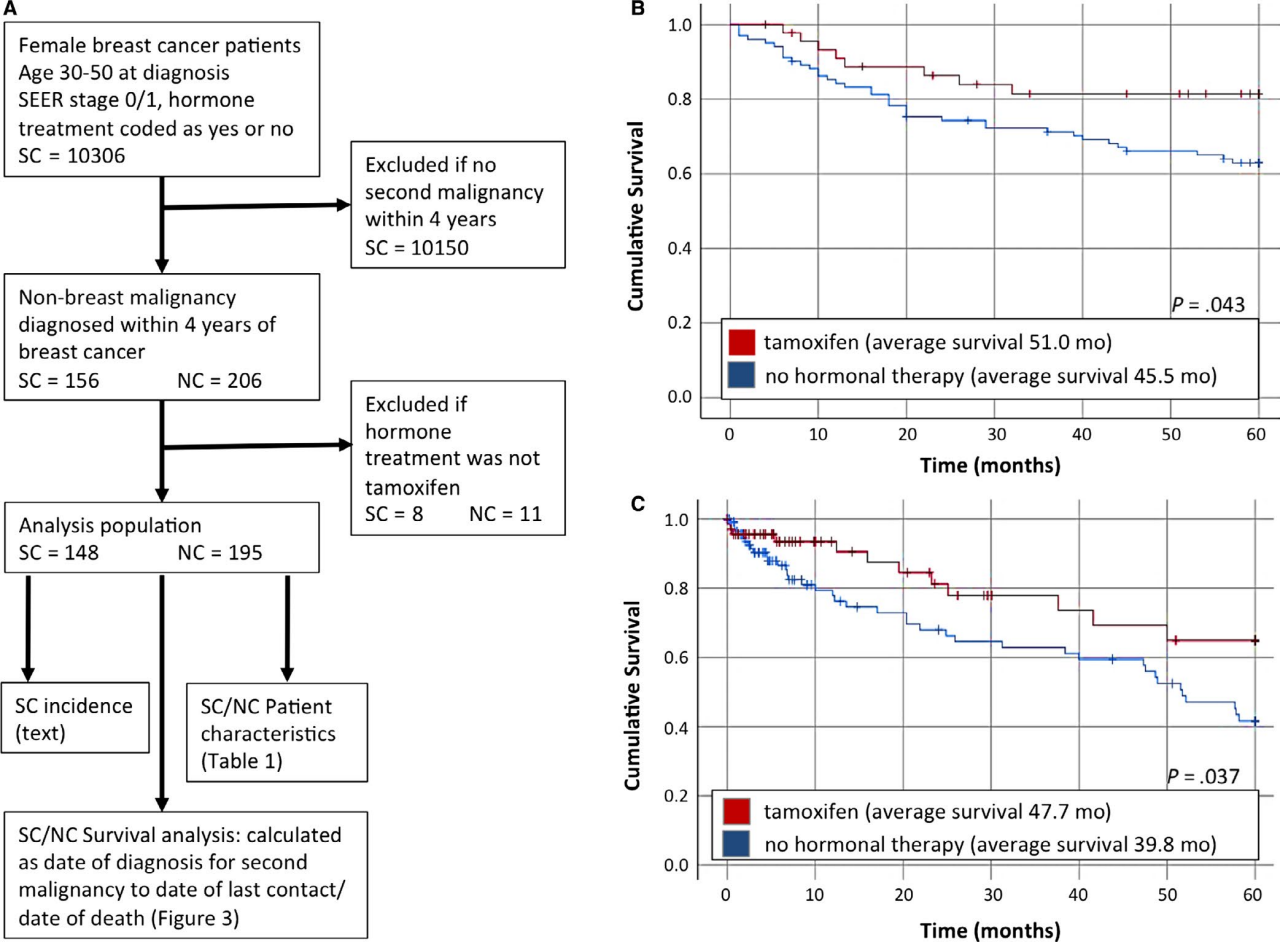
Effects of tamoxifen on nonbreast cancer malignancies. (A) Study design using North and South Carolina state central cancer registries (B) Kaplan‐Meier curves for second cancers in South Carolina, stratified by tamoxifen exposure. (C) Kaplan‐Meier curves of second cancers in North Carolina, stratified by tamoxifen exposure

Next, we evaluated whether the presence or absence of tamoxifen resulted in any trends relating to the second malignancies. There was no correlation between age, histology, primary site, and treatment modalities for the second cancers in the context of tamoxifen use (Table 1). Treatment modalities used in the original breast cancer were also evaluated for their potential downstream effect on the survival rates for the second cancers. Nearly all patients received surgery, as is appropriate for early stage breast cancer (at least 97% in every strata). Neither the use of chemotherapy for the original breast cancer, an indicator for triple negative histology or a larger tumor, nor the use of radiation, an indicator for lumpectomy rather than mastectomy, had a significant relationship to survival for the second cancer (Figure S2). Finally, we evaluated outcomes related to the second cancer using the study design as outlined in Figure 4A. Results show that survival outcomes for the second cancer were significantly improved in both South and North Carolina data sets, when the use of tamoxifen for an earlier breast cancer was assigned as the independent variable. The Kaplan‐Meier survival curves for the patients after their second (nonbreast) cancer, stratified by the presence or absence of tamoxifen, are shown in Figure 4B,C.

## 3 DISCUSSION

This study aims to address the only recently recognized clinical need to prevent PGCC offspring and cancer recurrence.^19^, ^20^, ^21^ A 2019 study demonstrated that ASAH1 is required for generation of PGCC progeny and we hypothesized that ASAH1 inhibition could be an effective maintenance therapy when a first course of treatment results in apparent cancer remission.^13^ Preclinical work has already demonstrated that remission‐free cures can be achieved in a prostate cancer mouse model when ASAH1 is inhibited during radiation therapy.^3^ The coincidence that tamoxifen, a commonly used antirecurrence drug used as a long‐term maintenance treatment in breast cancer, inhibits ASAH1 via an off‐target effect was unexpected but fortuitous. It has been suggested that tamoxifen possesses untapped anticancer potential as an ASAH1 inhibitor, independent of estrogen signaling.^18^ We specifically hypothesized that the hormone‐independent effect was due to inhibition of PGCC progeny and, in support, the current study demonstrates that tamoxifen blocked colony formation by PGCC in prostate cancer, melanoma, and glioblastoma cells (Figure 2). Tamoxifen, which we confirmed to inhibit ASAH1 activity, has also been shown to be active in combination with cisplatin against nonsmall‐cell lung cancer.^18^ This is in agreement with the 2019 study that showed a significant reduction in colonies following treatment of cisplatin‐generated A549 lung cancer PGCC with the ASAH1 inhibitor LCL521.^13^

Cancer patients are followed until time of death within cancer registries, which allowed us to trace the effect of tamoxifen use on outcomes for the subpopulation of women who developed a second (nonbreast) cancer within 4 years of initial breast cancer diagnosis. In general, individual hospitals would not have sufficient numbers to power the study we performed, and the National Cancer DataBase (NCDB) is de‐identified, which precludes tracing a single patient through multiple malignancies. Thus, in the United States, underutilized state‐level central registries are the most appropriate database to query for our intended analysis.^22^ Similar approaches have been used to study the effect of hormone therapy in breast cancer patients on lung cancer using the Manitoba and Geneva Cancer Registries.^23^, ^24^ However, we focused on refining this approach by specifically focusing on young women and tamoxifen use, while expanding to second cancers of all types.

Our study was necessarily limited by strict inclusion and exclusion criteria. We focused on young women with early stage breast cancer with the intent that these inclusion criteria would keep the comparison groups as similar as possible in terms of previous history of cancer, likely surveillance regimens, and general health. Women in this group have minimal mortality associated with the breast cancer diagnosis, and are routinely prescribed 5‐10 years of tamoxifen, if their cancer is positive for estrogen and/or progesterone receptors. However, we did not expect that our groups would be segregated purely according to hormone receptor status, since tamoxifen treatment may be waived for in situ breast cancer if treated with a double mastectomy or if women express the desire in becoming pregnant. Conversely, women with very low expression of estrogen or progesterone receptors may be prescribed tamoxifen at the medical oncologist's discretion. Unfortunately, in the United States, drug databases for patients in this age group are incomplete due to the fragmented nature of insurance, forcing us to rely on registry coding and supporting text fields in abstracts to assign patients to study groups.^22^ In countries with more integrated health care systems, it would have been possible to not only do larger queries of this type, but also to confirm that patients were filling prescriptions for the drug of interest. However, the strength of our study approach was bolstered by performing the same query on two different databases and selecting groups, which were otherwise as similar as possible, with tamoxifen exposure being isolated as a variable.

Similar to Bouchardy et al, who showed that use of antiestrogens did not impact the incidence of lung cancer, we found that use tamoxifen did not affect overall incidence of developing any second cancer.^24^ An increase or decrease in cancer incidence might have occurred if tamoxifen enhanced or reduced the generation of PGCC. Since ASAH1 was not required to form PGCC in response to stress, enzymatic inhibition of this enzyme was not expected to influence cancer incidence.^13^ We did hypothesize that the ability of tamoxifen to inhibit formation of PGCC progeny could have a positive effect on outcome for the second cancer. Indeed our results show that tamoxifen provides a significant survival benefit for second (nonbreast) cancers, which suggests that drug effects are not mediated through estrogen receptor signaling. Again our data is consistent with the study by Bouchardy and coworkers, who demonstrated that breast cancer patients receiving antiestrogens, primarily tamoxifen, had lower lung cancer mortality.^24^ Further support for the idea that tamoxifen exerts additional effects that are not estrogen‐dependent comes from studies of male breast cancer in which tamoxifen is significantly more effective than aromatase inhibitors.^25^ Similarly, combination of nanoliposomal ceramide with tamoxifen exerted antitumor effects in a hormone‐insensitive preclinical breast cancer study.^18^ Taken together, these data suggest that the therapeutic effect of tamoxifen beyond inhibiting estrogen receptor signaling could include an off‐target effect on ASAH1.

The context of estrogen signaling and ASAH1 requires careful consideration. In cancers that are not estrogen‐fueled, high ASAH1 levels likely reflect resilience due to stress adaptation. However, in estrogen‐fueled cancers such as female breast cancer, ASAH1 levels may be a proxy for estrogen dependence, since the ASAH1 promoter is a target of estrogen signaling.^26^ Since clinicians have effective means to block estrogen signaling, these treatments would indirectly inhibit ASAH1 expression though negative effects on the ASAH1 promoter, which would explain why high ASAH1 levels in breast cancer correlate with positive outcomes whereas the opposite has been reported for virtually every other cancer.^27^ The observation that targeting ASAH1 in estrogen receptor‐positive MCF7 breast cancer cells is sufficient to inhibit colony formation after radiation supports the idea that targeting ASAH1 downstream of the estrogen receptor is efficacious.^28^ Our analysis of carefully selected cancer registry data suggests that such a nonestrogen‐mediated effect could occur in a wide range of cancers.

**FIGURE 5:**
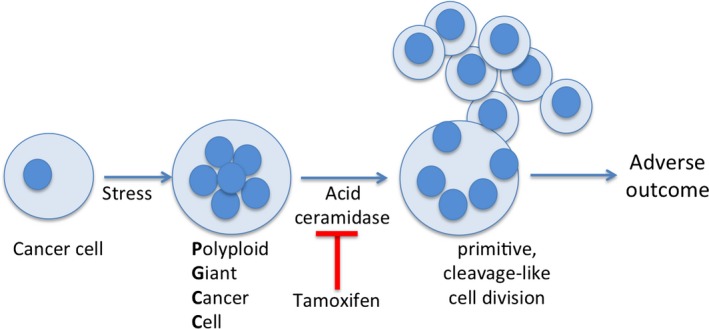
A model of cancer progression and intervention by tamoxifen. Exposure to stress can drive PGCC formation. Successful formation of progeny from PGCC leads to adverse outcomes due to increased metastasis, stemness, therapy resistance or a combination thereof. PGCC progeny formation is ASAH1‐dependent and can be inhibited by tamoxifen

The potential of tamoxifen efficacy has been tested in numerous clinical trials, including melanoma and glioblastoma, but success has been limited.^18^, ^29^, ^30^ The underlying reason for the limited success may lie in clinical designs that assumed tamoxifen would have activity against bulk tumor. A majority of cancer cells proliferate via mitosis but the co‐existing minority of PGCC that potentially produce progeny in a nonmitotic manner requires a different therapy approach. While many chemotherapies and radiation treatment interfere with mitosis, tamoxifen can be placed into its own category for prevention of PGCC progeny that arise nonmitotically. Our study suggests that future clinical trial designs should take into consideration the existence of a minority PGCC population that will evade antimitotic therapies and has the potential to repopulate the tumor (Figure 5). The presence of PGCC has been linked to poor outcomes in several cancers. In ovarian cancer the presence of PGCC in the stroma was associated with high metastasis.^11^ In colorectal cancers, an induction of PGCC and generation of daughter cells with strong migratory, invasive, and proliferative characteristics were observed following neoadjuvant therapy.^31^ In prostate cancer, where the typical mortality rate for the highest grade disease (Gleason score 9/10) is 5% at 2 years, the presence of PGCC was associated with a mortality of 37% at 8 months. If PGCC were observed after recurrence the mortality rate increased to 57% at 7 months.^32^ It has recently been proposed that the rare PGCC population within the tumor that withstands various stresses is equivalent to a keystone species on which the cancer ecosystem depends, which implies that targeting the process by which PGCC generate offspring may result in collapse of the tumor ecosystem.^20^ A brief report by Metzner and coworkers who describe long‐term remissions in metastatic melanoma if tamoxifen was provided as maintenance therapy following chemotherapy supports this idea.^33^ We therefore analyzed the 54 early stage melanoma cases in our data set. In patients not taking tamoxifen, 4/29 died late in year 5 of follow–up, whereas no deaths occurred in the tamoxifen group (0/25). Although the small patient numbers precluded reaching statistical significance, our data are supportive of the observations by Metzner et al. Going forward, it will be critical to identify markers for PGCC and develop a better understanding of the molecular requirements that allow the formation of PGCC progeny. We propose that the design of future clinical trials should include tamoxifen treatment in parallel with standard approaches such as chemo‐ or radiation therapy that promote the formation of PGCC. This combination is anticipated to not only target the bulk of diploid tumor cells but also reduce the primitive, cleavage‐like cell division from PGCC that drive relapse and recurrence.

## 4 METHODS

### 4.1 Cell culture and reagents

PPC1 prostate adenocarcinoma cells were obtained from Dr Dean Tang (Roswell Park Comprehensive Cancer Center); MEL624‐28 cells were obtained from Dr Michael Nishimura (Loyola University); U118 cells were obtained from Mediatech. Cells were cultured in RPMI (PPC1, MEL624‐28) or DMEM (U118) (Cellgro) supplemented with 10% FBS (Hyclone) and 1% antibiotic‐antimycotic solution (Gibco) and maintained at 5% CO_2_ in a humidified incubator. Cultures were not carried for more than 30 passages following authentication by STR (PPC1) or antigen expression/T cells reactivity (MEL624‐28) and were periodically verified to be free of mycoplasma using the MycoAlert PLUS kit (Lonza). Tamoxifen was purchased from Sigma. LCL521 was obtained from the Lipidomics Core at the Medical University of South Carolina.

### 4.2 Flow cytometry, recovery, and colony formation assays

To generate PGCC, cells were plated overnight and irradiated in a ^137^Cs γ‐irradiator (J.L Sheperd & Associates). The radiation dose for PPC1 and MEL624‐28 was 8 Gy and for U118 it was 12 Gy with PGCC appearing between 48‐60 hours. For flow cytometry, cells were plated 2 × 10^5^ on 60 mm plates, exposed to the indicated treatments, and analyzed on day 3 following trypsinization, fixation, and incubation with propidium iodide/RNAse. For recovery assays, cells were plated at 2 × 10^5^ on 60‐mm plates overnight and then left untreated or irradiated. After three days, media was changed and LCL521 added where indicated. LCL521 was refreshed every two days until parallel, untreated cultures approached confluency. Cells were fixed in ice‐cold methanol for 15 minutes (VWR), stained for 15 minutes with 0.05% crystal violet (Sigma), washed with tap water, and allowed to air dry. Cells were imaged using a 20× objective on a Zeiss Axiovert 200 equipped with an AxioCamMR. AxioVision software (version 4.8) was used to capture images. For quantification, the crystal violet stain was released with 10% acetic acid (Fisher Scientific) and signals quantified using a FLUOstar Optima plate reader (BMG Labtech). For colony formation assays, cells were plated 8 × 10^5^ on 100‐mm plates overnight and irradiated the next day. After three days, the cells were trypsinized and passed through a 20 filter (Pluriselect) to capture the PGCC. PGCC were plated sparsely on 6‐well plates and allowed to recover in the absence or presence of LCL521 or tamoxifen. Colonies were fixed with ice‐cold methanol, stained with crystal violet. Colonies containing at least 15 cells were counted.

### 4.3 Movies

The cells were imaged at 20× in the phase contrast channel using the BioTek Lionheart FX automated microscope. The images were captured using uniform exposure time every 15 minutes for 72 hours. Images captured in a single well were stitched together using Gen5 Image + version 3.05. Individual images were stacked to create movies, cropped, and scale bars added in ImageJ.

### 4.4 LC/MS analysis of sphingolipids

Cells were plated at 8 × 10^5^ in 100‐mm dishes overnight and either left untreated or irradiated the next day. On day 3, 5 µM tamoxifen was added for 5 hours, cells scraped into media, washed in PBS, and cell pellets were submitted to the Medical University of South Carolina Lipidomics Shared Resource for analysis of sphingolipids using by LC/MS.

### 4.5 Cancer registry query

Planned queries were reviewed by the institutional IRB, which determined the studies qualified as nonhuman subjects research. Staff at the state central registries performed queries on the databases and provided de‐identified data to investigators. Inclusion criteria were women age 30‐50 at diagnosis of an early breast cancer (SEER stage 0/1) with a subsequent nonbreast cancer arising within 4 years of the original breast cancer diagnosis. Women with unknown hormone treatment status for the breast primary or who received aromatase inhibitors were excluded. The North Carolina data set was found to have a high rate of censored data, which required additional exclusion, so that no censoring was introduced due to the lack of follow‐up time. Central registries typically link to state vital records and mortality data and the National Death Index on an annual basis to track mortality, and are generally complete through 2017 as of 2019, making 2012 diagnoses the preferred cutoff year for five‐survival analysis in this study. Therefore, cases with diagnosis of a second malignancy after 2012 were excluded. Patient characteristics analysis was performed with chi‐square testing, with groupings chosen to assure that no cell contained under five patients, per registry data use agreements.

### 4.6 Statistical methods

Two‐sided Student's t test, with alpha of 0.05 set as significant, was used for all comparisons of in vitro data. Chi‐square analysis was performed for each comparison in Table 1, with significance set as p values less than 0.05. Kaplan‐Meier analysis for Figure 4 was performed in SPSS 25 with a log‐rank test and significance assigned to *P* values under .05. Incidence rates for second malignancies were calculated, using the total number of eligible women (aged 30‐50 years at early stage breast cancer) in the South Carolina registry, stratified by hormone therapy. For calculation of incidence rate, the total number of patients receiving any hormone therapy was assumed to be similar to the number taking tamoxifen, as few patients required exclusion due to aromatase inhibitor use.

## CONFLICT OF INTEREST

JSN—The Medical University of South Carolina Foundation for Research has licensed LCL521 to SphingoGene, Inc JSN is the Chairman of the Board and Interim CEO of SphingoGene, Charleston SC. The remaining authors declare no competing financial interests.

## AUTHOR CONTRIBUTIONS

Shai White‐Gilbertson and Christina Voelkel‐Johnson conceptualized the study. Shai White‐Gilbertson led the investigation with support by Ping Lu, Christian Jones, and Arabinda Das. Deborah Hurley and Stephanie Chiodini curated data and generated software code for analysis of patient data. James S. Norris, Joe R. Delaney, and Christina Voelkel‐Johnson acquired funding and provided supervision. Shai White‐Gilbertson and Christina Voelkel‐Johnson validated all data and wrote the manuscript.

## Supporting information

Fig S1

Fig S2

Movie S1

Movie S2

Movie S3

## Data Availability

The data that support the findings in patients are available from the Cancer Registries of North and South Carolina. Restrictions apply to the availability of these data as they are only available from the respective Registries and cannot be shared by the corresponding author. The data that support the in vitro data of this study are available from the corresponding author upon reasonable request.
