# Supplementary figures and images for: Tamoxifen is a candidate first‐in‐class inhibitor of acid ceramidase that reduces amitotic division in polyploid giant cancer cells—Unrecognized players in tumorigenesis
Source: Cancer Med. 2020 Mar 5;9(9):3142–52. doi: 10.1002/cam4.2960 (PMC7196070; doi:10.1002/cam4.2960)

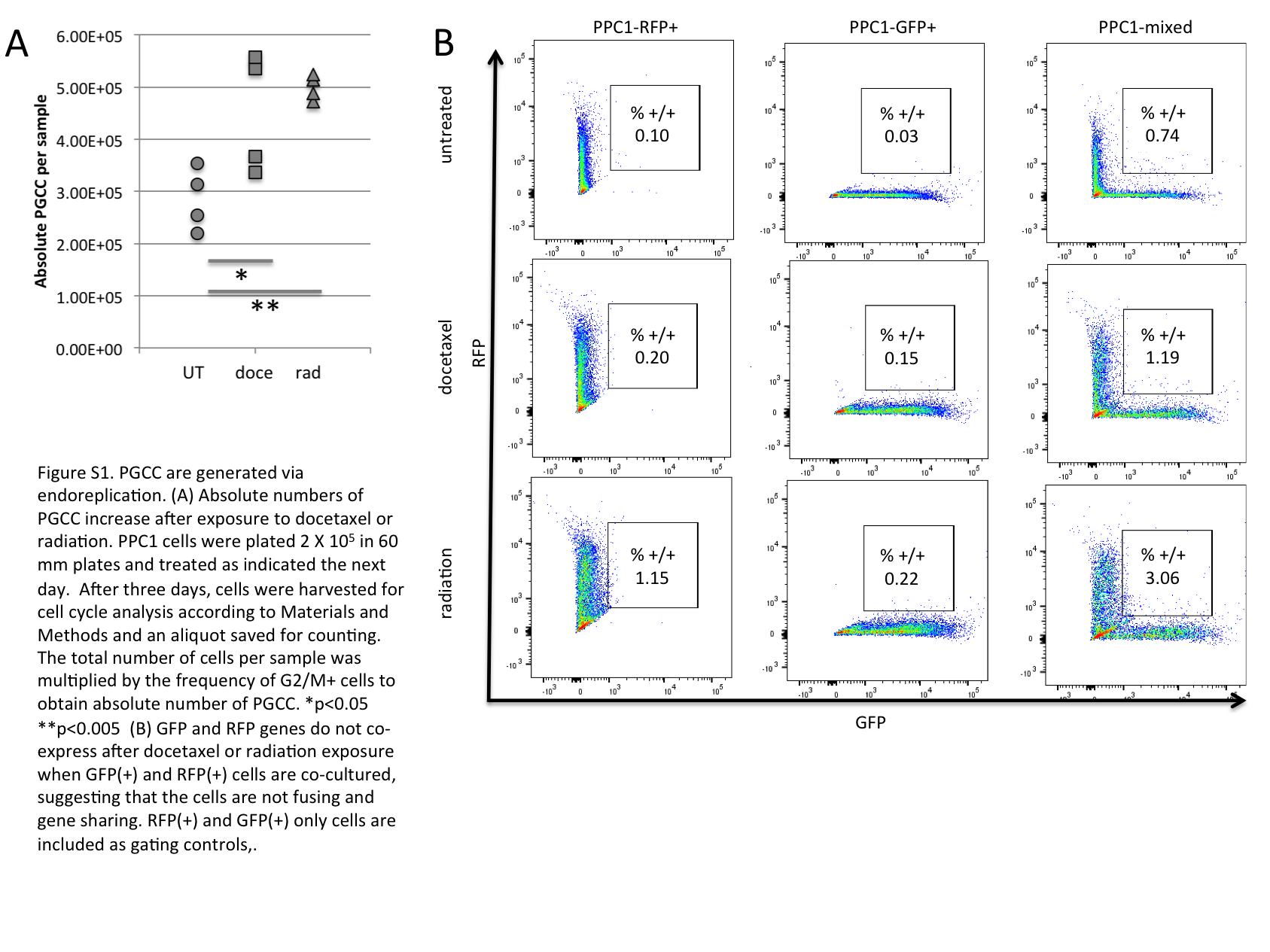

Supplement: Supplementary file 1 — Fig S1 [file CAM4-9-3142-s001.tiff]

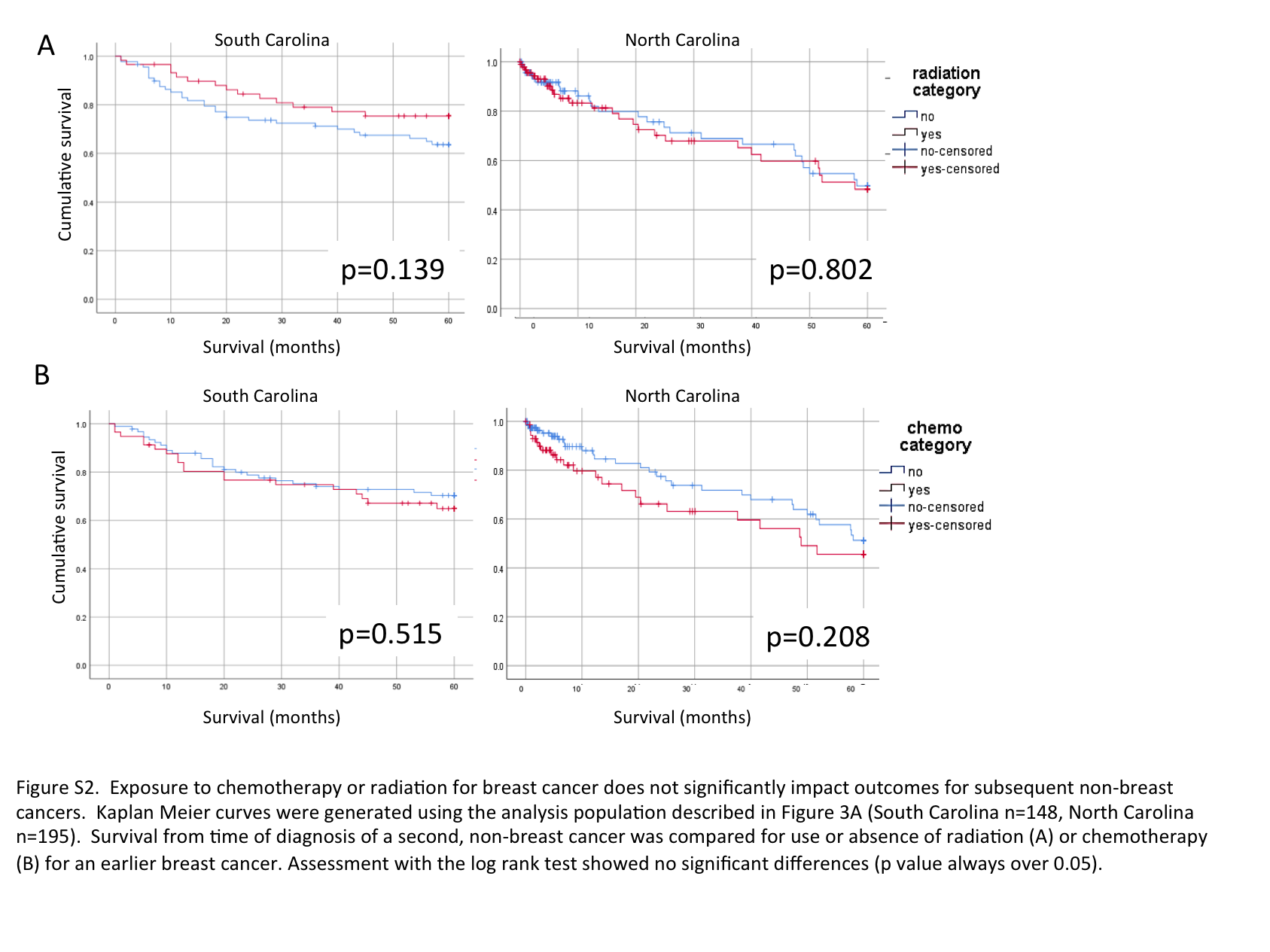

Supplement: Supplementary file 2 — Fig S2 [file CAM4-9-3142-s002.tiff]
